# 2000 Years of Grazing History and the Making of the Cretan Mountain Landscape, Greece

**DOI:** 10.1371/journal.pone.0156875

**Published:** 2016-06-09

**Authors:** Isabelle Jouffroy-Bapicot, Boris Vannière, Virginia Iglesias, Maxime Debret, Jean-François Delarras

**Affiliations:** 1 Laboratoire Chrono-environnement, UMR 6249 CNRS, Université Bourgogne Franche-Comté, 25000 Besançon Cedex, France; 2 Maison des Sciences de l'Homme et de l'Environnement, USR 3124 CNRS, Université Bourgogne Franche-Comté, 25000 Besançon, France; 3 Morphodynamique Continentale et Côtière, UMR 6143 CNRS, Université de Rouen, 76821 Mont-Saint-Aignan Cedex, France; Chinese Academy of Sciences, CHINA

## Abstract

Understanding the processes that led to the recent evolution of Mediterranean landscapes is a challenging question that can be addressed with paleoecological data. Located in the White Mountains of Crete, Asi Gonia peat bog constitutes an exceptional 2000-years-long sedimentary archive of environmental change. In this study, we document the making of the White Mountains landscape and assess human impact on ecosystem trajectories. The paleoenvironmental reconstruction is based on high-resolution analyses of sediment, pollen, dung fungal spores and charcoal obtained from a 6-m core collected from the bog. Multiproxy analyses and a robust chronological control have shed light on anthropogenic and natural processes that have driven ecological changes, giving rise to the present-day Mediterranean ecosystem. Our results suggest that sediment accumulation began during the transition from the Hellenistic to the Roman period, likely due to watershed management. The evolution of the peat bog as well as vegetation dynamics in the surrounding area were linked to past climate changes but were driven by human activities, among which breeding was of great importance. Charcoal analysis reveals that fire was largely used for the construction and maintenance of sylvo-agropastoral areas. Pollen data allow the identification of three main vegetation assemblages: 1) evergreen oak forest (before ca. 850 AD), 2) heather maquis (ca. 850 to 1870 AD), 3) phrygana/steppe landscape. Rapid changes between phases in vegetation development are associated with tipping-points in ecosystem dynamics resulting from anthropogenic impact. The modern ecosystem did not get established until the 20^th^ century, and it is characterized by biodiversity loss along with a dramatic drying of the peat bog.

## Introduction

Understanding the relationship between societies and their environment is of great importance for the sustainable management of ecosystems [1]. Retrospective studies, from local to regional scale, are essential in this regard because they help us decipher the evolution of social-ecological system dynamics over time [2]. The Mediterranean basin is of especial interest, inasmuch as it is a particularly sensitive area currently threatened by environmental change.

Despite the difficulties in delimiting a singular ‘Mediterranean area’, the Mediterranean basin has a set of similar environmental characteristics, such as climatic cycles, common land uses (e.g., breeding, olive cultivation) and land management (e.g., burning, terrace), which allows its definition as a homogeneous region [3]. In addition, it is cradle to the birth of many ancient large and powerful civilizations.

Since the early 20^th^ century, two main hypotheses have been proposed to explain the effect of human societies on the Mediterranean landscape. The theory of the “Lost Eden” addresses the concept of degradation and desertification of the landscape resulting from land-use; conversely, human impact has also been proposed to shape and maintain landscape diversity [4]. Testing these hypotheses requires the elucidation of the timing and features of past environmental changes in relation to changes in land use. Although disentangling the relative importance of human-versus climate-induced changes in Mediterranean landscapes has been a challenging goal in numerous paleoecological studies, from Western to Eastern Mediterranean (e.g., in Spain [5, 6], in Italy [7–9], in Greece and Turkey [10–16], high-resolution multi-proxy analyses remain scarce. These kinds of analyses allow not only to reconstruct the evolution of the landscape but also to identify thresholds in the resistance and resilience that may lead to rapid environmental change.

**Fig 1:**
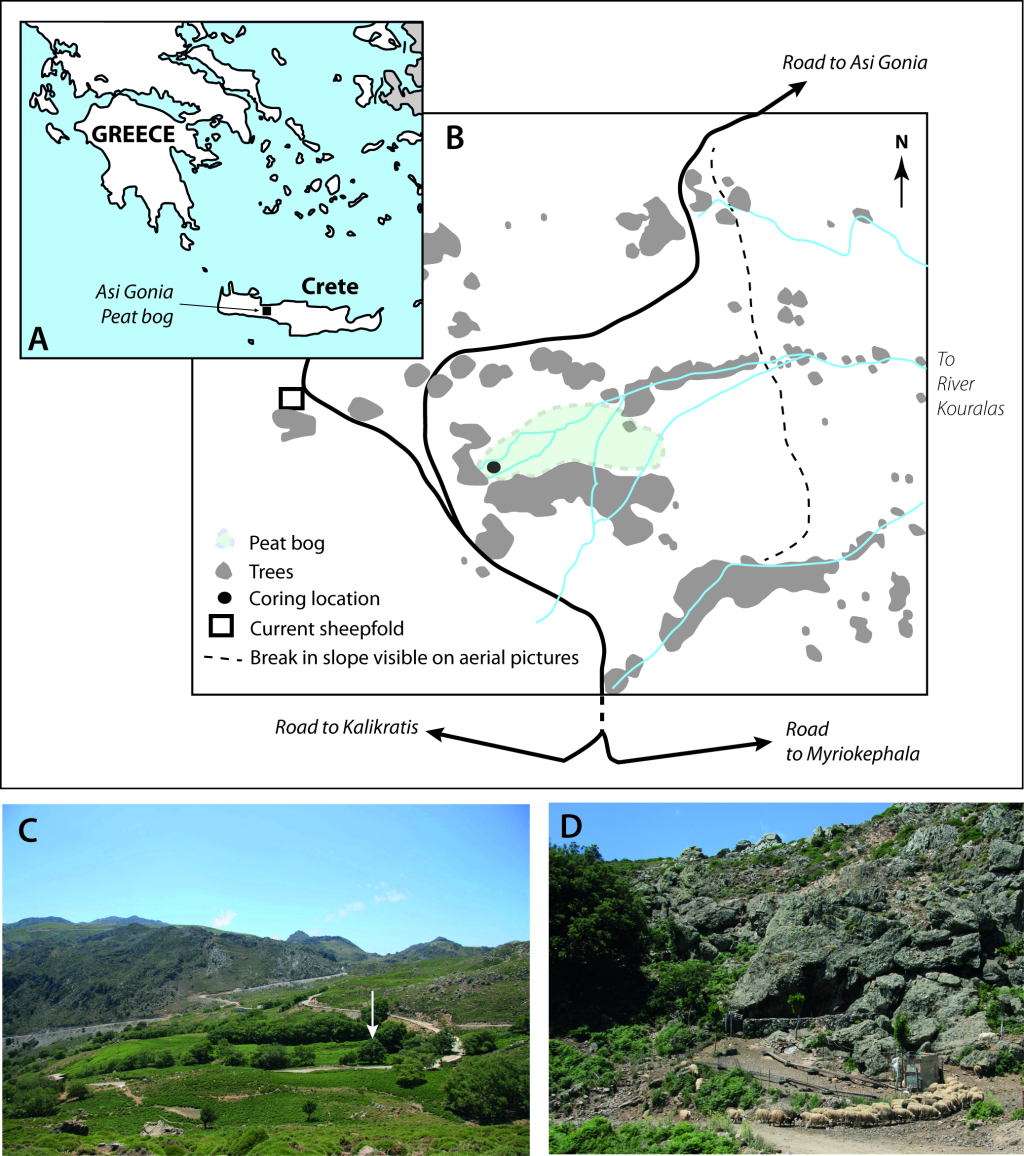
(A) Location of Asi Gonia peat bog in Crete, Greece; (B) map of the peatland showing the coring location; (C) Photo of the peatland area. The white arrow indicates the coring site; (D) Photos of the dry stone sheepfold and rock shelter located near the peat deposit; (photos: I. Jouffroy-Bapicot, T. Pedrotta, May 2012).

Crete is the largest Greek island in the Aegean Sea (Fig 1). Located in the northeastern biogeographical zone of the Mediterranean [17], it is liable to Atlantic, Saharan, eastern European and western Asian climate influences and lies on the cross road of west- versus east and north- versus south-central Mediterranean climate processes [18–21]. This island is of great interest for ecological studies due to its very sharp gradients: a one-day-road trip allows going from mountains with permanent snow to sea shores with endemic palm-trees, crossing forests, deserts, steppes and maquis.

Discovered by scientists in the 1980’s, Asi Gonia peat deposit is the only peat bog on the island and a rare continental sedimentary archive in the southeastern Mediterranean Basin. A previous palynological study [22, 23] highlighted the unprecedented potential of this natural archive to investigate landscape and biodiversity evolution under changing climates and human impact over the last two millennia. Our objective is to explore deeper this exceptional potential through high-resolution and up-to-date multi-proxy analyses. Specifically, our work aims to document the shaping of White Mountains landscape and assess land-use legacy on the present state of the ecosystem.

## Background

### Regional setting

Asi Gonia peat bog is located in western Crete, at the eastern edge of the White Mountains (Lefka Ori), which reach 2453 m elevation on Mount Pahnes (Fig 1). If the climate of Crete is typical Mediterranean, this mountainous part of the Island is wetter, subject to the influence of the westerlies and with a proportion of winter precipitation in the form of snow [24]. Located at an altitude of 780 m elevation between the villages of Asi Gonia, Kallikratis and Myriokephala, the peat deposit is a spring-fed formation at the head of one of the feeder streams of the River Koularas. The substratum and watershed of the bog are formed by phyllitic schist and altered mesozoic limestone [22]. The vegetation surrounding the bog is mainly composed of phrygana. This typical undershrub Cretan formation composed of hemispherical, spiny and often aromatic dwarf shrubs, such as *Sarcopoterium spinosum* and *Genista acanthoclada*, develops in places exposed to dry conditions, and is highly adapted to browsing pressure and burning [24]. Steppes with some stands of taller scrub and woodland are found at a larger scale. These features have not significantly changed since the end of the 1990s [22]. We observed in different sections of the watershed some patches of *Pteridium aquilinum* underlining areas with the deepest soils [24]. *Platanus orientalis* and some *Castanea sativa* border the site. *Pteridium aquilinum* widely dominates the peat deposit at present; the open land with *Carex* and *Juncus* is restricted to a small western part. Fielding et al. [24] published a description of the bog vegetation at the beginning of the 21^th^ century. The dramatic drying of the site observed at that time is still in process especially as streams that fed the wetland are largely captured by herders. Breeding is currently important in the area. A dry stone sheepfold close to a rock shelter is still used (Fig 1) and flocks of sheep and goats are widespread both in the watershed and in the close surroundings of the peat bog itself. Localized burning places of the phrygana’s under shrubs were observed in the area during our coring campaign in 2011. Terrace crops, especially olive-trees, are cultivated at a slight lower altitude.

### Archeology and history from the Roman period to Present Day

In 69 BC, Crete became part of the Roman Empire. The city of Gortyn, located on the southern coastline, became the capital of the joint province of Crete and Cyrenaica. Even if Romanization of the island remained low, Crete prospered under Roman rule [25]. After the fall of the Western Roman Empire, the First Byzantine (324–824 AD) is considered as a period of great prosperity in Crete, with the construction of aqueducts, farms and massive Christian basilicas, without significant disruption to the previous planning. After one century of raids on the coastal areas of the island, the Saracens, Muslims from Andalusia, achieved the conquest of Crete in 824 AD. The Arabs founded a new capital on the northwestern coastline, Chandax, present-day Heraklion. Thereafter, and during 150 years, this island represented a strategic economic and military base in the Muslim world. In 961 AD, Crete was re-incorporated to the Byzantine Empire and divided into a feudal system that would prevail until Modern Times. After the end of the fourth Crusade and the fall of the Byzantine Empire, Crete entered in the gain of the Venetian Republic in 1204 AD. The "mother city" organized land management and production of its colony. Venetians became landowners or merchants on the island and Venetian economic policy organized wheat and wine exportation [26]. The latter became particularly important and lands for grape cultivation were progressively gained at expense of other crops. According to archeological and historical data, the 13^th^ century and particularly the end of the period seems to have been times of prosperity, while during the first half of the 14^th^ century revolts and epidemics of bubonic plague affected the island. Thereafter, the end of the Venetian period experienced again times of prosperity. After 24 years of battles, the Ottoman conquest of Crete was completed in 1669. Under the Turkish administration, major changes in island agriculture took place, mainly concerning the increase of grain cultivation, and plantation of olive trees for oil exportation at the expense of vineyards. During the 18^th^ century, Crete entered in a period of trouble, anarchy, epidemics and wars that let the island bled dry [25]. In 1913, it joined Greece and experienced inside and outside wars. Thus, during the two last millennia, Crete’s history has been closely linked to the outside world: Roman, Byzantine, Arab, Venetian and Turkish empires [27], and its economy and land use have been under the rule of the dominants.

## Material and Methods

### Field work, radiocarbon dating and age-depth modelling

**Fig 2:**
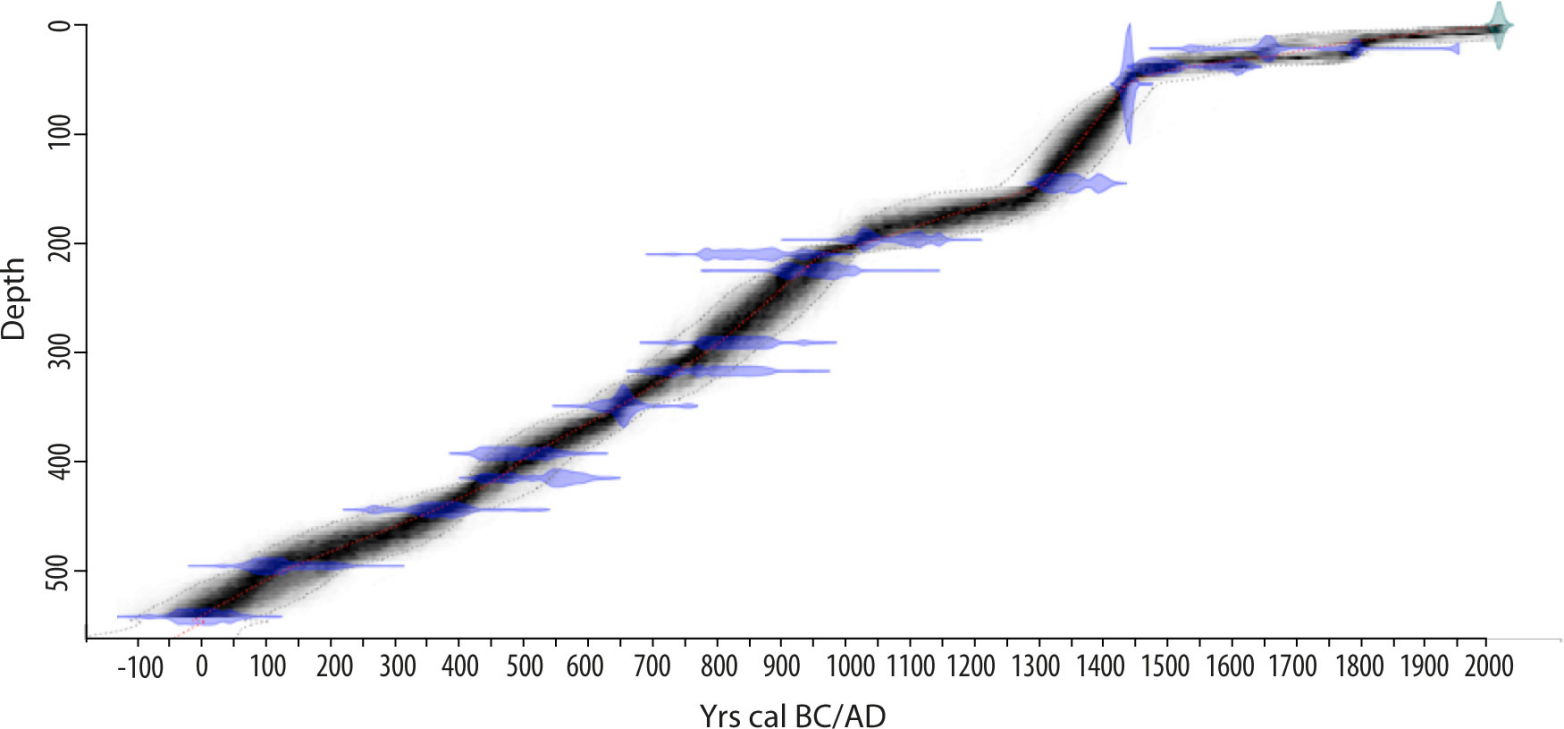
Age-depth model of the AG11 sequence (Asi Gonia, Crete, Greece) developed with Bacon 2.2 (acc.mean = 3.5 (yr cm^-1^) and acc.shape = 0.50; [29]).

After a survey of sediment accumulation thickness all over the site, twin-cores were retrieved with a GYK “Russian” corer from the deepest part of the peat blanket (35°14”55 N, 24°16’38 E), near its western edge (Fig 1A & 1C; the Greek Institute of Geology and Mineral Exploration (I.G.M.E.) authorized fieldwork and core analyses). The total master core is 577 centimeters long. The sequence is composed of alternating facies of organic sediment with variable degrees of peat decomposition (more or less fibrous) and with also varying mineral content. A total of 15 samples, mainly on terrestrial macro-remains, were submitted for AMS radiocarbon dating (Table 1). Calibration of the ^14^C dates was based on the latest IntCal13 calibration curves [28] and age-depth modelling was performed with Bacon 2.2. The prior distribution for sediment accumulation had an expected value of 3.50 years cm^-1^ and a shape of 0.50 [29] (Fig 2).

**Table 1:** AMS radiocarbon dates from cores AG11 from the Asi Gonia peat bog (Crete, Greece). Calibration has been done using the most recent IntCal13 calibration curves [28].

Sample ID	Depth (cm)	Material	Lab Code	^14^C Age	Cal BC/AD 2σ
P1 0–100 21–23	21,5	Bulk	Poz-63612	245±30	1523–1950 cal AD
P1 0–100 38–39	38,0	Wood	Poz-43404	375±25	1448–1631 cal AD
P2 50–150 4–6	54,0	Seeds	Poz-63613	465±30	1411–1462 cal AD
P2 50–150 95–96	145,0	Wood	Poz-4814	600±30	1297–1408 cal AD
P2 150–250 61–62	196,5	Charcoal	Poz-43735	980±30	994–1154 cal AD
P2 150–250 74–76	210,0	Wood	SacA 28620	1165±30	773–966 cal AD
P1 200–300 34–36	225,0	Macro-remains	Poz-63616	1085±35	892–1017 cal AD
P1 300–400 12–14	291,0	Wood	Poz-4813	1190±30	723–944 cal AD
P2 250–350 84–86	317,0	Wood	Poz-4815	1225±35	688–886 cal AD
P1 300–400 70–72	349,0	Wood	Poz-63615	1375±30	609–683 cal AD
P2 350–450 62–63	392,5	Acorn	Poz-43407	1560±30	421–563 cal AD
P2 350–450 84–86	415,0	Wood	Poz-4817	1520±30	428–609 cal AD
P2 450–550 15–17	444,0	Acorn	Poz-4818	1675±35	253–501 cal AD
P1 490–590 24–26	494–6	Macro-remains	Poz-63614	1885±30	61–218 cal AD
P1 490–590 71–72	541–542	Bark	Poz-43408	2000±30	83 cal BC-70 cal AD

### Sedimentology

**Fig 3:**
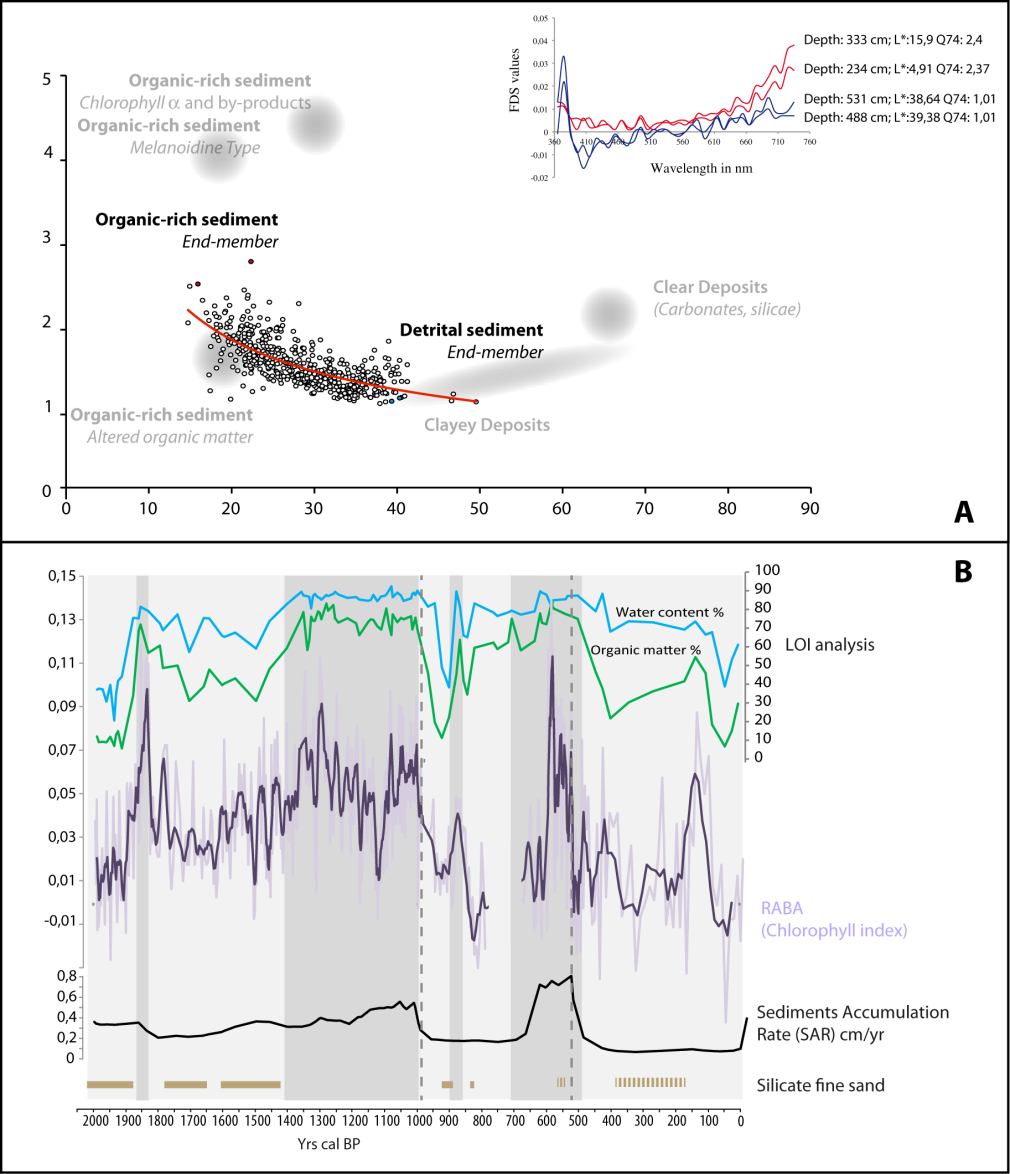
**Sedimentological analyses of the AG11 core (Asi Gonia, Crete, Greece);** (A) Q7/4 diagram [32] based on spectrophotometric measurements. The abscissa axis represents the 700/400 μm ratio (main slop of the raw spectrum) and the ordinate axis the lightness of the sediment L* (White/Black). Usual sediment product values and AG11 sedimentary end members are indicated; (B) from top to bottom: water content (%), LOI 550 (estimate of organic matter content; %; Heiri et al., 2001), RABA Chlorophyll Index from spectrophotometric measurements, SAR—sediment accumulation rate from the Age-Depth model (Fig 2), and presence/absence of silicate fine sand in the peat facies.

Sediment analyses involved Loss On Ignition (LOI) and spectrophotometry measurements (Fig 3). LOI aims to measure the organic and mineral content of sediment samples, allowing the characterization of the material and the inference of processes and environmental conditions involved in its deposition [30]. All LOI analyses were carried out in a “Nabertherm controller B170” with digital temperature display and thermostatic temperate control. The samples were previously dried at 105°C overnight and cooled to room temperate in a desiccator before any measurements were made. After oven-drying of sediment samples of constant weight, organic matter was combusted to ash and carbon dioxide at a temperature between 500 and 550°C [31].

Spectrophotometric analyses aim to quantify the color of sediments (e.g. [33, 34]), providing a high resolution (cm-scale), quick and non-destructive data measurement of sediment reflectance. Using the Q7/4 diagram [32], we assessed the sedimentological potential of spectrophotometric measurements to characterize sedimentary facies and reconstruct sedimentological dynamics. For this study, spectrophotometry data were obtained with a Minolta CM 2600d. The illuminant was D65, which corresponds to average day light, and the wavelengths belong to the extended visible domain from 360 to 740 nm. The measurement aperture was set to 8 mm and the step of measurement to one centimeter. By taking the optical lightness L* (White/Black) *vs* 700/400 ratio (main slop of the raw spectrum), it is possible to distinguish the different sedimentary end members that constitute the sediment. In addition, we used a down core index, the RABA Chlorophyll Index [35] in order to trace the “Chlorophyll a and by products” concentration in the sediment. The RABA Chlorophyll Index has been interpreted as an indicator of autochthonous paleoproductivity in lacustrine sediment [35].

### Charcoal analysis

For charcoal analysis, 297 contiguous samples of 2 cm^3^ were retrieved by volumetric displacement at 1 or 2-cm intervals. The samples were soaked in a 10% NaOH solution for 24 hours for peat digestion and then in a 30% H_2_O_2_ solution for the same time to bleach non-charcoal organic material and thus make charcoal identification easier [36]. As we aim to reconstruct the local fire history, quantification of charred particles was performed after sieving the samples with a 150 μm mesh [37, 8]. Charcoal identification was performed with a binocular microscope at 50× magnification and based on criteria defined in the literature [38, 39]. Charcoal particle counts were then converted to charcoal concentration (CHAC; particles cm-^3^).

Paleofire signal analysis was based on the decomposition method initially described by Long et al. [40], with the aid of CharAnalysis 0.9 [41]. The main steps of the signal decomposition are the following: the macro-charcoal sedimentary record was first interpolated to a median sampling resolution of 6 years and transformed into macro-charcoal accumulation rates (CHAR; particles cm-^2^ year^-1^); then, to eliminate the slowly varying component or background signal of the charcoal record, influx values were smoothed with a robust locally weighted regression type function (Lowess) with a 50-year window [42, 41]. Background charcoal influx (BCHAR) may be driven by several sedimentary processes (e.g., deposition of reworked particles from littoral sediment; [43]). Alternatively, it may be linked to fuel availability and characteristics [44, 45] or regional fire activity [37]. Since subaerial peat is likely to better reflect atmospheric transport processes than lakes [46], at Asi Gonia, we expect to have low background levels resulting from water transport, especially as the very small watershed is not drained by any permanent rivers.

**Fig 4:**
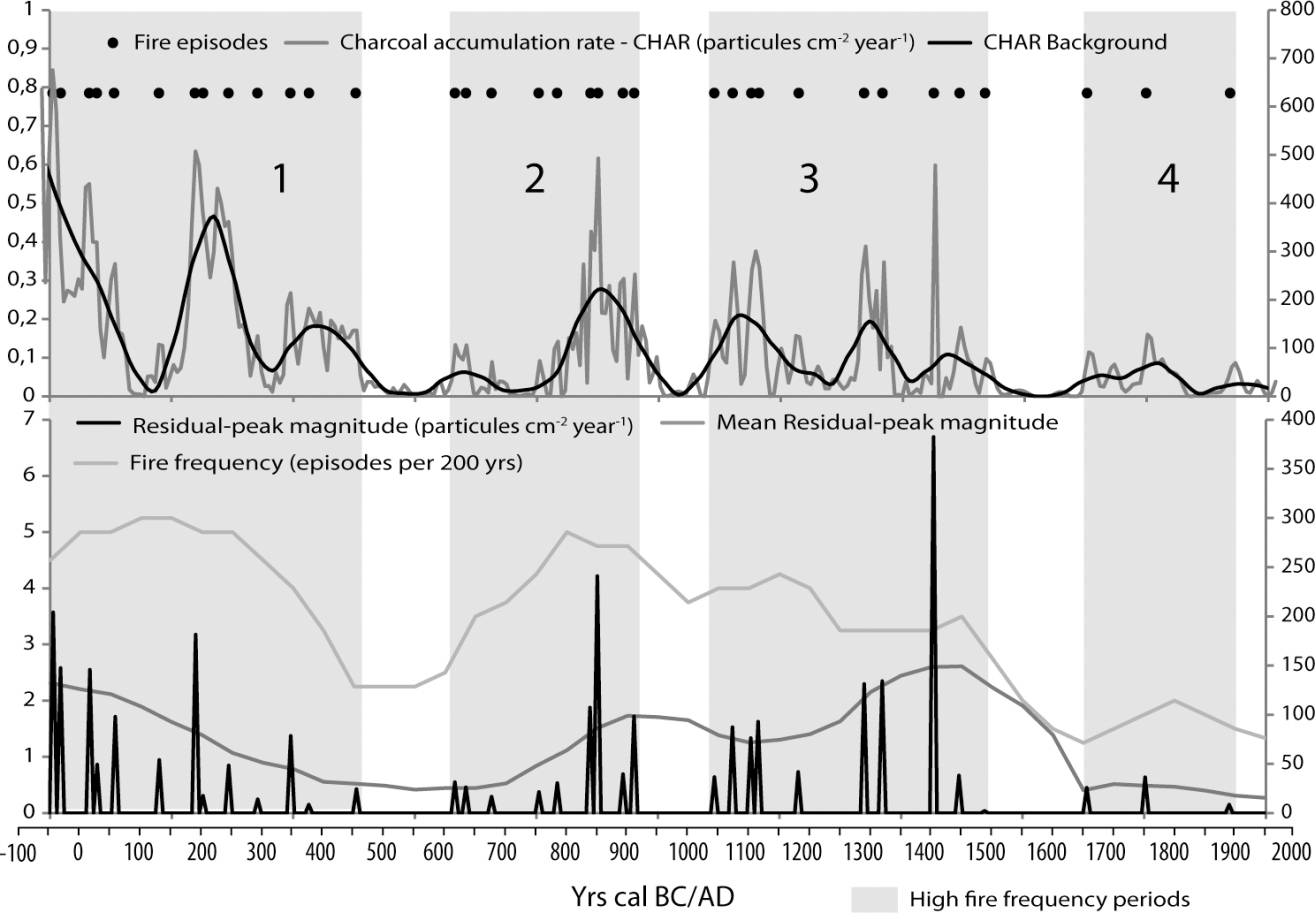
Macro-charcoal data from the AG11 core (Asi Gonia, Crete, Greece) and reconstructed fire regime parameters: fire episodes, fire-episodes frequency, fire-episodes magnitude (CHAR residual-peak), and mean fire-episodes magnitude.

The difference between charcoal influx and the background component defines the peak component [42, 47]. Peaks are assumed to represent fire episodes in the local or micro-regional area around the site. Estimation of the peak component or fire episodes allows the reconstruction of paleofire regime parameters: the magnitude of fire peaks was used as an approximate (qualitative) indication of fire intensity or the area burnt [41]; and fire frequency was calculated as the total number of fires within a 200 year window (Fig 4). Mean fire intervals (MFI) were calculated by dividing the years between the first and the last fire episode in a given period by the number of episodes-1.

### Pollen and NPPs analysis

**Fig 5:**
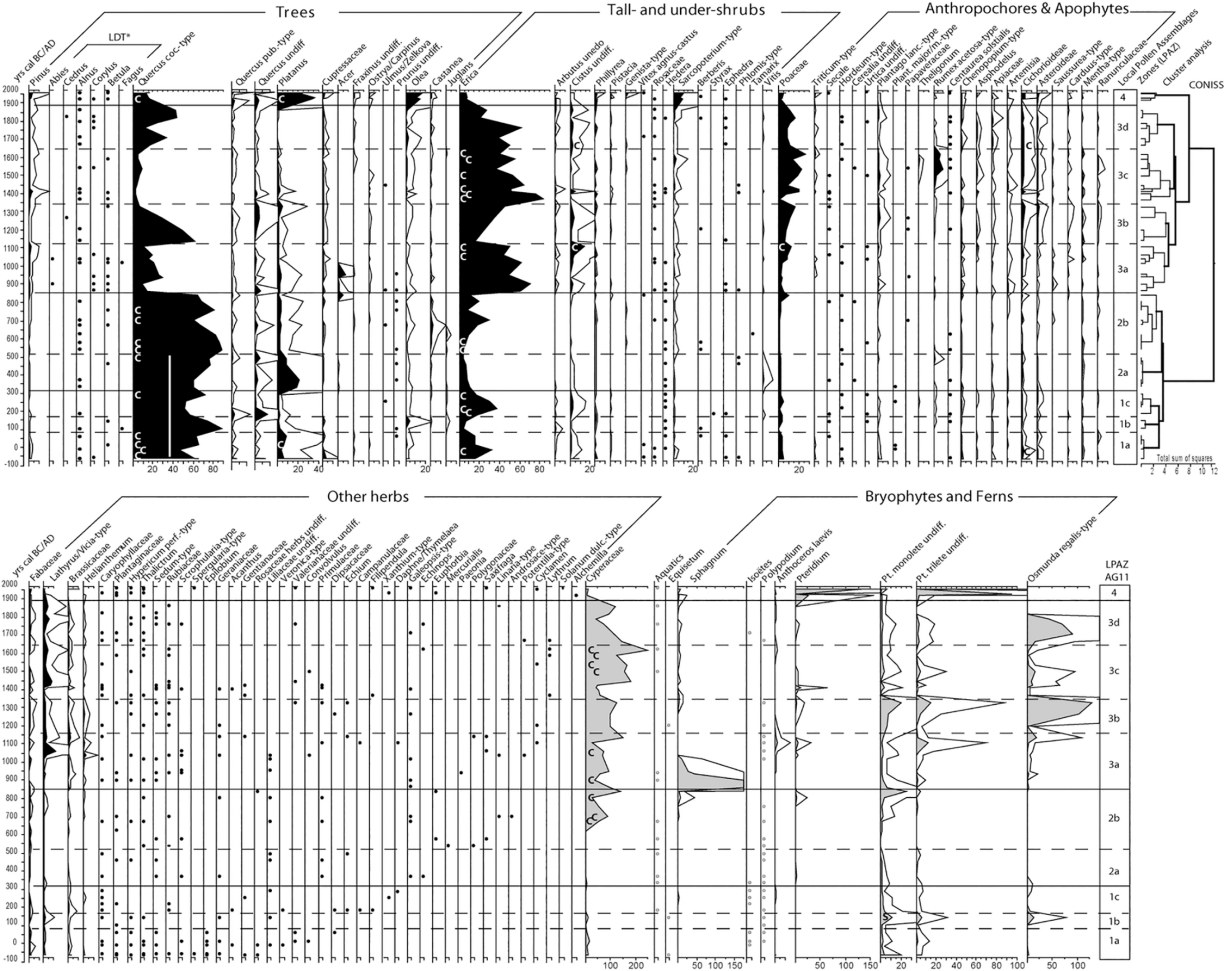
Complete percentage pollen and spore diagram from the AG11 core (Asi Gonia, Crete, Greece). Taxa excluded from the TLP sum are in grey; LDT* = long-distance pollen (Pollen of pines and trees absent in the modern ecosystems of Crete is likely to result from long-distance transport onto the island [53]); c = clump of pollen grains indicating the proximity of the plant of origin; the white bar indicates the presence of oak macro-remains (leaf, acorn) in the peat.

2 cm^-3^ samples were taken at a mean interval of ca. 50 years for pollen and non-pollen palynomorph (NPPs) analyses. Samples were prepared according to standard techniques for pollen analysis [48]. Pollen grains were identified with the aid of keys, photographs [49, 50] and reference to the modern pollen collection at the Chrono-environment Laboratory of Besançon. Nomenclature followed Beug [51]. The identification of *Triticum*- and *Hordeum*-type pollen was performed according to Beug [51] as well. It was based on grain morphology, namely grain, pore and annulus size, and the exine ornamentation observed with the aid of phase contrast microscopy. Pollen percentages were based on the sum of dry-ground vascular plant pollen (i.e., total terrestrial pollen or total land pollen; TLP), which, in all cases, exceeded 500 grains. Fern and bryophyte spores, aquatic plants and Cyperaceae were excluded from the TLP. Relative values of pollen were calculated as a percentage of the TLP sum using Tilia, and the pollen diagrams were constructed with TGView [52] (Fig 5).

**Fig 6:**
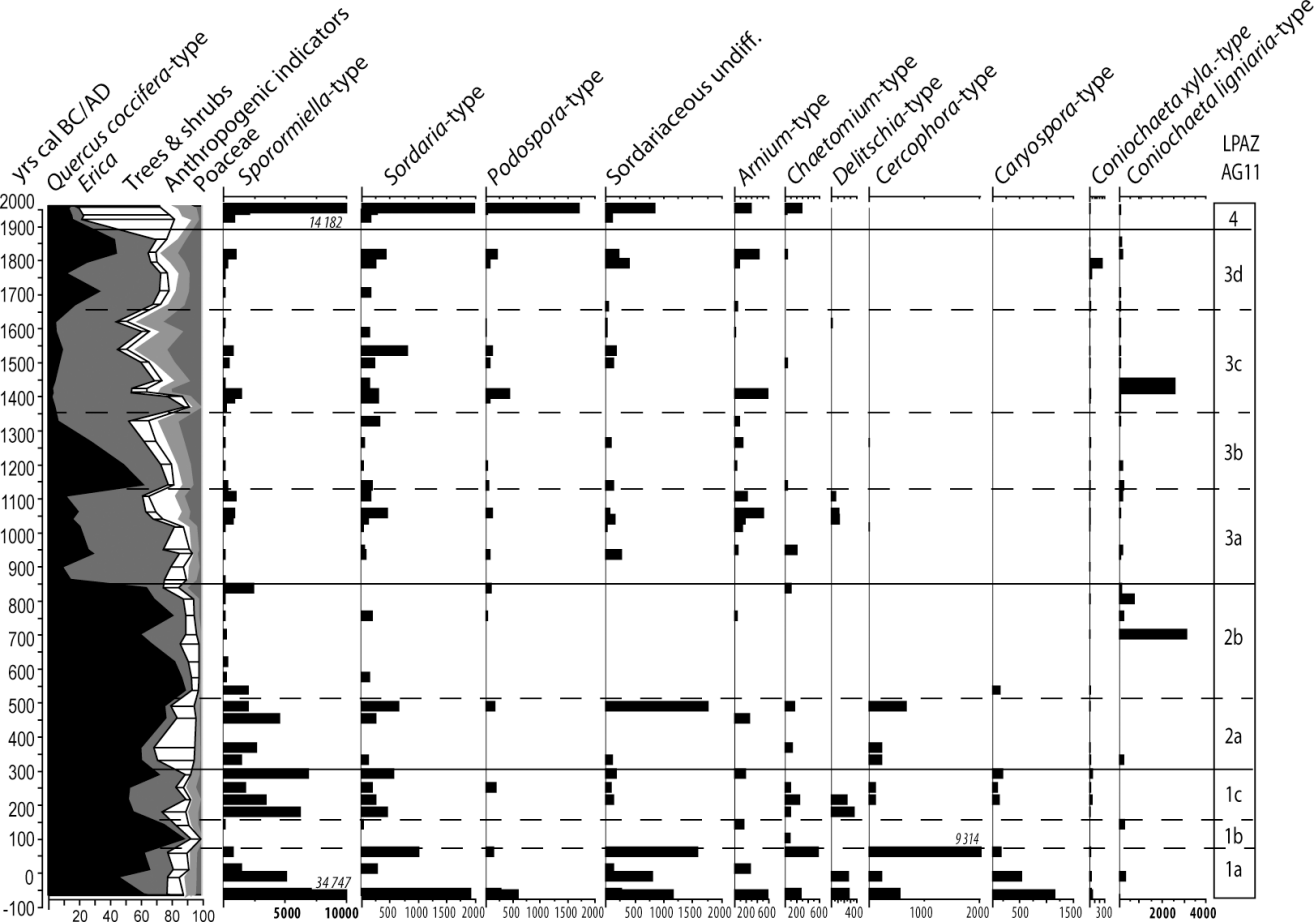
Saprophytic and dung fungal spore taxa and summary pollen diagram from AG11 core (Asi Gonia, Crete, Greece).

In addition to pollen analysis, NPPs were identified and counted using published descriptions by [54–57], and particular attention was paid to dung fungal spores, a widely used proxy of past presence of herbivores [58, 59]. In this paper, the main coprophilous fungal spores are employed [58] in the reconstruction of pastoralism history (Fig 6). Complete NPPs analysis will be the topic of a future study. *Lycopodium* tablets [60] were used to estimate pollen and NPPsconcentrations (grains cm^-3^) and accumulation rates (grains cm-^2^ yr^-1^). A minimum count of 350 *Lycopodium* spores per sample was required for the assessment of fungal spore concentration [61]. The zonation of the pollen diagram was performed with the aid of the module CONISS included in Tilia, based on the square roots transformation of stratigraphically constrained TLP counts.

**Fig 7:**
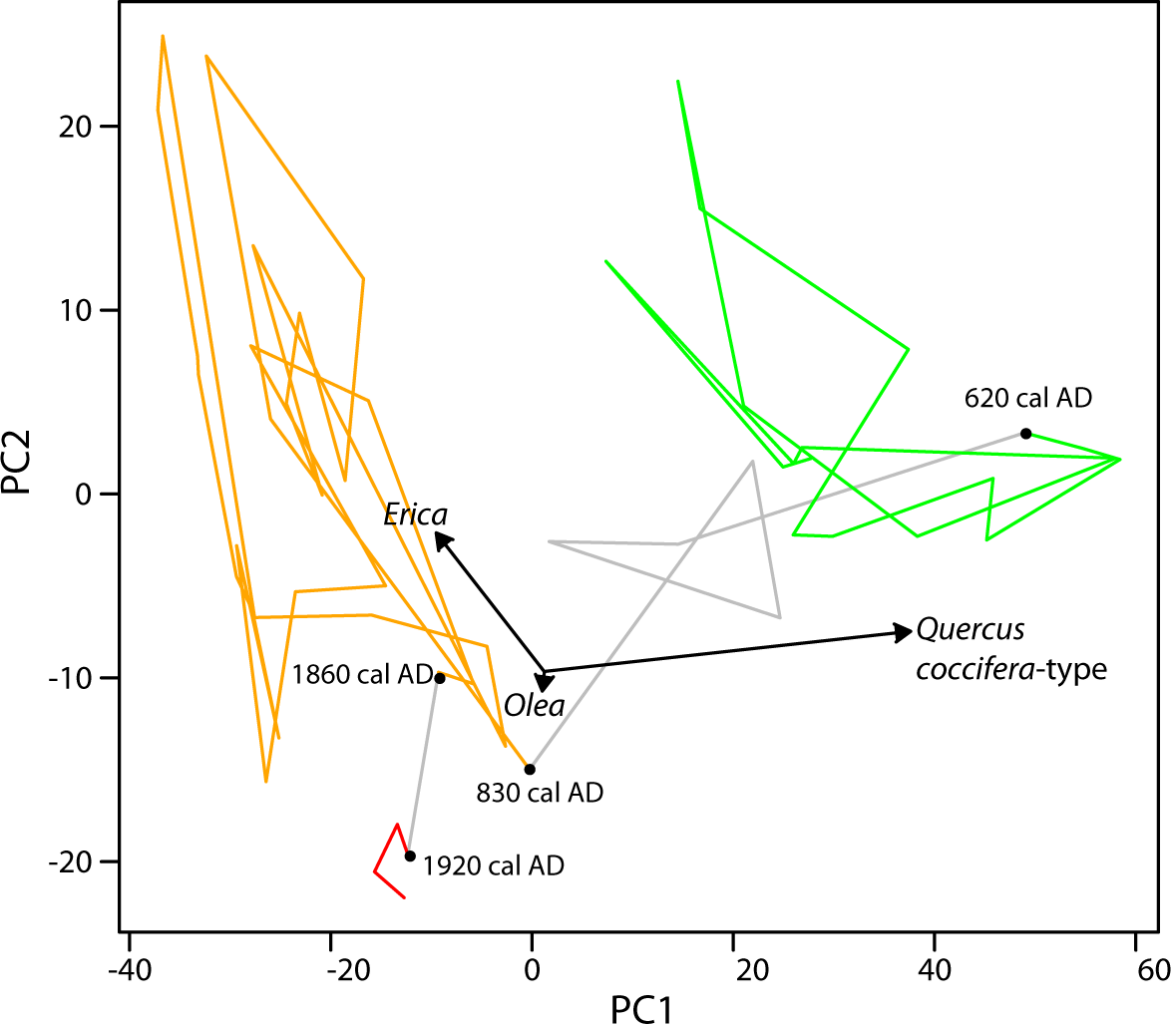
Principal component analysis (PCA) of the terrestrial and regional pollen data grouped in vegetation zones (in color). Principal components one (PC1) and two (PC2), eigenvectors (arrows), and temporal boundaries of the main vegetation zones (black dots) are shown. Wetland taxa (*Sphagnum*, ferns spores, Cyperaceae and *Platanus*) have not been included.

Temporal changes in vegetation composition were summarized by performing principal component analysis (PCA) on the terrestrial pollen percentage dataset. The calculation was done by eigenvalue decomposition of the correlation matrix using the Stats package in R (R Core Team, 2015). Principal components one and two explain 75% and 14% of the total variance, respectively (Fig 7). Vegetation zones were defined by maximization of within-group similarity and among-group dissimilarity.

**Fig 8:**
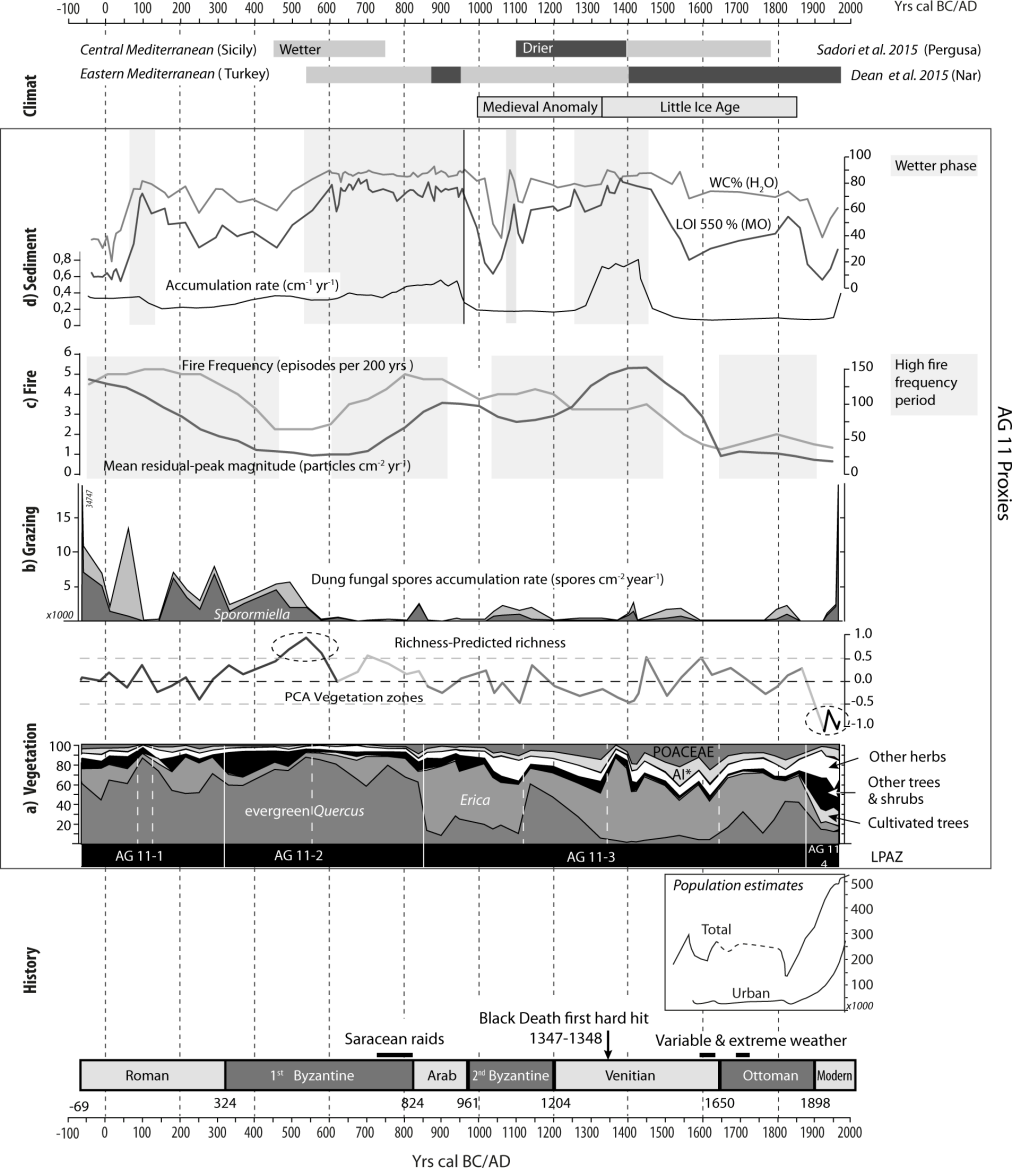
Synthesis of key environmental proxies from the AG11 core (Asi Gonia, Crete, Greece), Cretan historical information (compiled from [25]), and Mediterranean recent Late Holocene climate data [7, 67]. a) Vegetation: summary pollen diagram and richness-evenness curve; b) Grazing: fungal spore accumulation rate; c) Fire: frequency and mean peak magnitude inferred from charcoal accumulation rate (CHAR) analysis; d) Sediments: LOI analysis and sediment accumulation rates.

In order to explore past biodiversity patterns, we estimated temporal trends in palynological richness (Fig 8). Palynological richness, defined as the number of taxa per sample at constant pollen sum [62], is a frequently used indicator of biodiversity in paleoecology. However, and despite being considered a pretty robust method, the results may be influenced by the total pollen sum, pollen evenness (i.e., relative proportion of pollen types in a sample) and the distribution of pollen types in the sediment [63–65].

To account for these limitations without much information loss, we calculated the adjusted palynological richness for the time series as proposed by [66]. Specifically, we developed a linear model of palynological richness as a function of evenness in which (1):

$$APS_{\left(t\right)}=0.43\;E_{\left(t\right)}+1.49$$ (1)

where APS_(t)_ is the adjusted palynological richness of a sample at time t and E_(t)_ is the palynological evenness of the same sample based on taxa exceeding 0.3% of the TLP.

The difference between the palynological richness calculated using rarefaction to 500 grains and the estimated APS constitutes a biodiversity index that incorporates both the number of species and their abundance. Positive deviations are interpreted as additions of taxa to the system and negative deviations as richness loss.

## Results

### Lithostratigraphy and sediment

The chronology of the core, based on 15 AMS radiocarbon dates, covers slightly over 2000 years without any hiatuses (Fig 2). Peat accumulation was continuous during the first millennium and more variable afterwards. In the Q7/4 diagram, the values of brightness (L*) vs 700/400 ratio were organized between two major end-members that remained constant throughout the record (i.e., “organic rich deposit” and detrital with “clayey deposit”; Fig 3A). This result shows that the composition of the sediment, and the peatland processes were homogenous through time, with oscillations between a wet-organic phase and a drier-more mineral one. These oscillations can also be traced through variations in the water content, residual organic matter and RABA index curves, which are positively correlated. In all cases, large positive values indicate wet-organic phases (Fig 3B). The base of the core, which corresponds to the beginning of sediment accumulation, is the least organic sample of the sequence. Overall, the bottom part of the sequence (0–500 cal AD) is characterized by rather mineral and dry conditions, interrupted at around 100 AD by a significantly more humid and organic event lasting ca. 50–100 years. From 500 until 950 AD, the local conditions switched to a more humid and organic phase that led to an increase in sediment accumulation rates (SAR). A more abrupt transition occurred at 950 AD, characterized by a strong decrease in SAR, sedimentary organic matter and water content, and the RABA index. This new dry phase of the peatland lasted around 350 years (until 1300 AD) when an increase of the RABA index, along with related proxies of organic matter productivity and accumulation, reflect a second phase of peat accumulation associated with wetter environmental conditions. Since 1500 AD, the peat bog has recorded a third mineral phase and experienced dryer conditions, which, with the exception of a short humid phase between 1750 and 1850 AD, became progressively more intense until the 19^th^ century.

### Charcoal and fire regime

The frequency and magnitude of fire episodes inferred from the sedimentary macrocharcoal content quantification allow identifying four main paleofire regimes (Fig 4). The first one lasts from the onset of the sequence to around 450 AD. It is characterized by high fire frequencies (up to 5 events 200 years^-1^) that decrease after 300 AD. This period is also characterized by a continuous decrease in the magnitude of fire episodes. No fire episode has been detected between 450 and 600 AD. A second phase of fire occurrences is recorded afterwards, marked by a return to high fire frequencies and an increase in the magnitude of the fire episodes until 900 AD when the signal drops abruptly. No fire episodes are registered between 900 and 1050 AD either. The third phase is characterized by moderate to very high fire episode magnitude, particularly at the end of the period, and a slow decrease of fire frequency spanning 400 years (until 1450 AD). No fire episodes are detected between 1450 and 1650 AD. Finally, the last fire regime phase, which covers the last three and an half centuries, corresponds to few remote and weak fire episodes (i.e., very low fire frequency and fire signal magnitude). Despite the seasonal burning currently used by shepherds to favor the palatability of the phrygana’s dwarf shrubs (authors’ observation), very few charcoal particles were conserved in the upper part of the record, suggesting that this kind of practice is almost invisible in the sedimentary archive if performed outside the watershed.

### Pollen, fern spores and fungal remains

The complete pollen and spores diagram is displayed on Fig 5. Selected fungal spore taxa discussed in this paper are shown in Fig 6.

#### LPAZ AG11-1 (ca. 0–300 AD)

The first pollen zone (AG11-1) is dominated by *Quercus coccifera*-type (from 50 up to 80% of the TPL) followed by *Erica*-type (reaching 30% of the TPL) and *Cistus*-type. During this period, the proximity of oak trees and heather to the peatland is corroborated by the presence of macro remains, acorns and leaves of evergreen oak and pollen clumps of *Quercus coccifera*-type and *Erica*. *Platanus* is well represented in subzone 1a but decreases in subzone 1b. *Cerealia*-type grains are scarce but constant, and so are fungal remains, of which saprophytic and coprophilous taxa are particularly well represented. A sharp increase in evergreen oak pollen characterizes subzone 1b, while shrub and herb pollen as well as fungal spores dramatically decrease. Zone 1b corresponds to a rise in the *Olea* curve. *Juglans* and *Castanea* appear at the time and percentages of fern spores of undifferentiated Pteridophyta and *Osmunda regalis-type* temporally rise. The dynamics of pollen, ferns and fungal spores at the bottom of this zone (1c) are similar to those of LPAZ 1a.

#### LPAZ AG11-2 (ca. 300–850 AD)

LPAZ 2 begins with a rise in *Platanus* pollen followed by a gradual increase of *Quercus coccifera*-type percentages. Shrubs, herbaceous taxa and anthropogenic indicators decrease. In LPAZ 2b, *Quercus* evergreen pollen is still largely dominating albeit it decreases around 650 AD, when small peaks of *Olea* and *Castanea* are observed. Fungal remains slightly decrease but diversity remains comparable to that of the previous zone.

Subzone 2b is characterized by the long-lasting development of Cyperaceae. The presence of agro-pastoral indicators remains low, but rises at the end of the zone, and so do wild Gramineae and fern spores (mainly monoletes spores). *Sphagnum* spores, which show occasional occurrences before, become better represented. Fungal remains are present but coprophilous and/or saprophytic taxa become rare or totally disappear. Dung fungal spores of *Sporormiella*-type increase again at the end of the zone.

#### LPAZ AG11-3 (ca. 850–1900 AD)

The transition between zones 2 and 3 displays a dramatic and sustained decrease of *Quercus*, along with an increase of *Erica* and, to a lesser extent, of other shrubs, mainly *Cistus* and *Arbutus unedo*. A continuous curve of *Acer* pollen, which appears at the end of the previous zone, rises in subzone 3a. Agro-pastoral indicators and herbaceous taxa favored by human activities remain scarce. A sharp rise of *Sphagnum* spores is observed in the first half of the zone, followed by an increase in the diversity and amount of fern spores (*Anthoceros laevis*, *Osmunda regalis* and other Pteridophyta) as well as dung fungal spores.

An important rise of *Quercus coccifera*-type (up to 50%) characterizes the transition from subzone 3a to 3b. However, this rise is not long-lasting and percentages of evergreen oak pollen decrease sharply, falling under 15% at the end of the zone, when *Erica*-type pollen increases. A pronounced increase in Poaceae, herbs -particularly herbs favored by human activity, such as *Plantago lanceolata*-type- and a growing abundance of fern spores, mainly *Osmunda regalis*-type, are registered. Conversely, the amount and diversity of fungal remains become very low.

This dynamic is punctually interrupted at the transition between zones 3b and 3c, around 1300 AD. *Erica*–type pollen rise anew, at the expense of a strong decrease of wild Poaceae, other herbs and *Sporormiella*-type ascospores. Following this event, subzone 3c corresponds to the main rise of herbaceous taxa through the diagram, including Poaceae and Cyperaceae. In addition, *Triticum*-type pollen is more abundant than before (even if percentages do not exceed 1%), human-related plants reach their maximum and weeds are more important and diversified than ever. With the exception of a peak of dung fungal spores at around 1500 AD, fungal remains are rare.

Subzone 3d displays an overall increase in *Quercus coccifera*-type percentages, despite the irregular nature of the curve. An inverse pattern characterizes the *Erica* curve. Globally, herbaceous taxa and human indicators decrease. The first half of the zone is marked by an important peak of *Osmunda regalis*-type, which abruptly and definitively disappears at the end of the zone (ca. 1900 AD).

#### LPAZ AG11-4 (ca. 1900–2011 AD)

The uppermost pollen zone is characterized by a clear disruption in the pollen diagram, with a dramatic fall of *Erica*, a continuous decline of Poaceae and Cyperaceae and a strong depletion of herbaceous taxa. In contrast, *Platanus*, *Olea*, and *Sarcopoterium*-type clearly rise. The increase in *Pteridium* spores is very pronounced, and *Sporormiella* spores along with other dung fungal spores are numerous in the upper section of the core.

PCA suggests that a large proportion of the temporal variability in vegetation can be explained by changes in the relative proportion of *Quercus coccifera*-type and *Erica* and to a lesser extent *Olea* (Fig 7). Based on the floristic composition of the pollen samples, three very distinct vegetation zones can be distinguished (i.e., before 600 AD, 800–1850 AD and 1900 AD to present), with one transitional zone between 600 and 800 AD.

Prior to 600 AD, arboreal taxa were abundant in the assemblage and, as described for pollen zones *LPAZ AG11-1* and *LPAZ AG11-2*, the period was characterized by oscillations in the relative proportion of *Quercus* in the landscape and *Platanus* in the wetland. The transitional period ended with a sharp decline in *Quercus* observed at ca. 500/580 AD. Between 800–1850 AD (pollen zone *LPAZ AG11-3*), the ecosystem was dominated by non-arboreal taxa and characterized by centennial-scale advances of *Erica* at expense of Cyperaceae. The increase of *Olea* at ca. 1850 AD culminates in the establishment of present-day vegetation.

Palynological richness is quite stable during the two first PCA phases but presents a rather high value between 300 and 800 AD with a maximum around 500–600 AD. A significant loss of biodiversity is recorded after ca 1850–1900 AD (Fig 8).

## Discussion

### Peat bog origin and evolution

**Fig 9:**
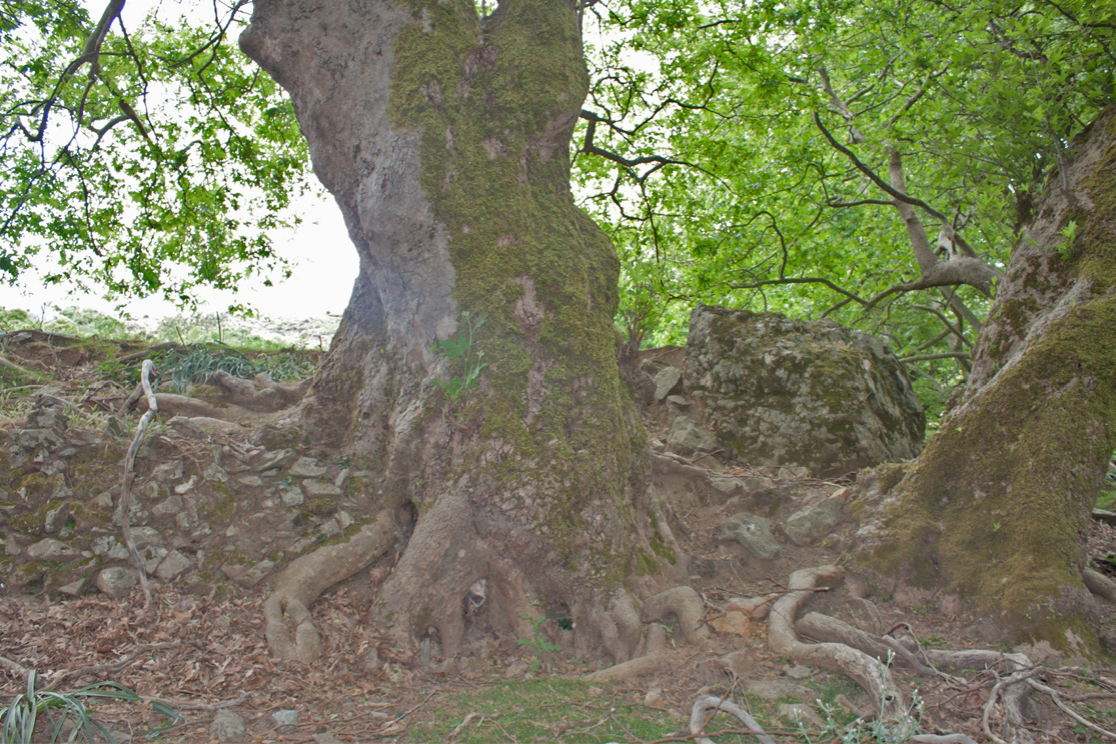
Photograph of a big plane tree growing on a dry-stone wall bordering the peat deposit (photo: T. Pedrotta, May 2012).

The origin of peatlands can be linked to natural features, such as climate or tectonic activity, or to human impact on the hydrological cycle [68]. Based on the analysis of their longer sediment core, Atherden & Hall (1999) attributed the formation of Asi Gonia peat bog to large tectonic activity recorded between 200 and 500 AD. However, the bottom of our core dates back to ca. 50 BC, suggesting that the onset of sediment accumulation is older than previously proposed. Given that no significant tectonic activity has been recorded at the time, we hypothesize that the origin of the peat bog is of anthropogenic nature because: 1) drier-than-before conditions prevailed in the eastern Mediterranean during the last 4600 years [18], indicating that the natural water balance is not likely to have been favorable for peatland formation; 2) human impact, especially grazing and burning, has been quite strong from the beginning of the sequence; 3) there are undated remains of human constructions such as terraces, buildings, and walls in the watershed and on the peat bog itself (Fig 9). In the Mediterranean, human induced peat accumulation in basin mires has also been observed in Northern Italy [69]. Although the origin of Asi Gonia peat bog is very likely to be human-induced, further field work is required to test the hypothesis and elucidate whether it was intentional (e.g., to make a water reservoir), or the indirect result of disturbance associated with watershed management.

In the light of sedimentological changes coupled with local vegetation and fungal remains dynamics, two main phases in the Asi Gonia peat bog evolution may be distinguished (Fig 8 top panel). The first phase lasts between 50 BC and 1000 AD and follows a classic pattern of a peat bog evolution, with a minerogenic onset phase followed by growing organic accumulation [68]. Local grazing and burning did not hamper this process, but the decrease of local land use around 100 AD and after 550 AD might have favored this dynamic. From 500/550 AD onward, humid and organic components of the peat deposit reached their maximum, when a shift to more humid-than-before conditions was recorded elsewhere in the Mediterranean (e.g., at 450 AD in Sicily [7] and at 550 AD in the Eastern Mediterranean, at the Nar lake in central Turkey, where this shift was especially strong [67]). Thus, the Asi Gonia peat bog sedimentary dynamics attest here to the role of large-scale climate conditions in local environmental processes. Along with this evolution, the local decrease in evergreen oak, which is illustrated by the disappearance of oak macro-remains in the peat (Fig 5), favored light demanding species. As a result, Cyperaceae spread after 650 AD, followed by a sharp rise in *Sphagnum* (Fig 8).

An abrupt shift to drier conditions occurred in the wetland around 950–1000 AD. Although this shift is concomitant with the beginning of the 2^nd^ Byzantine period, paleoecological bio-indicators did not record any changes in human activity at the time that could be linked to this process. In addition, recent studies suggest wetter climate prevailed in the Eastern Mediterranean after 950 AD [20, 67]. We therefore infer that watershed-scale processes probably drove local drier conditions. Afterwards, sediment variations became more pronounced and difficult to link to regional climate changes. On the wetland, fern assemblages dominated, and registered some large peaks of *Osmunda regalis* spores (Fig 5). Between ca. 1300 and 1500 AD wetter local conditions were registered in the peat bog. It is worth noting that this time period corresponds to the beginning of the little ice age (LIA). However, it is difficult to link this local process to global climate change, especially since the first phases of LIA in the North-Eastern Mediterranean corresponded to drying process (e.g. [16]. The acceleration of peat accumulation is probably related to local humid conditions along with the strong decrease of human pressure that occurred between 1300 and 1350 AD. Thereafter, all indicators point to drier and more detrital conditions, which reached their maximum after ca. 1850 AD. Palynological data suggest that, at the time, the wetland experienced a dramatic decrease in the local biodiversity of ferns and fungal spores as well as loss of *Sphagnum* that is supported by field observations [24].

### 2000 years of grazing, burning and land-use history

The main changes in the local landscape revealed by zonation of the pollen diagram (LPAZ) closely coeval with chrono-cultural limits (Fig 8). Pollen analysis by Atherden & Hall (1999) pinpointed the correlation between vegetation changes and human history, whereas dung fungal-spore analysis of AG11 peat core illustrates the predominant role of pastoralism during the last 2 millennia (Fig 6). *Sporormiella*-type, one of the most reliable indicators of herbivore presence and the most frequently used proxy of pastoral activities [58], is recurrent all over the sequence. In the top-layer of the peat core, high accumulation rates of dung fungal-spores reflect the present-day presence of flocks of sheep and goats in the watershed, up to the edge of the peatland.

Charcoal analysis indicates that fire activity has been mainly connected to pastoralism throughout the record, especially during the first 500 years when fire episode occurrences are associated with the rise of grazing indicators (Fig 8). During the Roman period, both dung fungal spores and charcoal data indicate intense human activity, and the likely access of animals to the wetland. During the transition from the Hellenistic to the Roman Imperial period, historical data such as treaties and poetry attest to 1) the importance of milk and derived products of milk in the diet and 2) specialized pastoralism and transhumance in the Cretan mountains [70]. Fire events were not recorded during a few decades around 100 AD and dung indicators suggest lower grazing pressure in the surroundings of the bog, while evergreen oak forest cover reached its maximum density/ extension. At Asi Gonia, the Late Roman period and the assimilation of Crete into the first Byzantine Empire did not cause large changes in the ecosystem: environmental proxies show that grazing and burning remained locally important until ca. 500 AD, despite a gradual decrease of fire frequency and mean peak magnitude.

Between 450 and 600 AD, there is no evidence of fire, and indicators of local grazing sharply decline until 500 AD and remained low during the following centuries. It is possible that wetter climate, as recorded in the peatland and elsewhere in the Mediterranean around 500 AD, made this mountainous area less attractive at that time. A new fire regime got established at the turn of the 7^th^ century AD but dung fungal spores remained scarce.

Around 820–850 AD, while Crete fell under Saracens rule, a brief increase in dung indicators occurred in association with a local maximum in fire magnitude and frequency (Fig 8). Climate conditions, as inferred from sedimentological proxies, remained humid and, although the peatland was not affected by this event, led to an unprecedented depletion of the evergreen oak forest. Human populations found shelter in the mountainous areas of the island in order to outrun Saracen’s raids and occupation of the coastal cities [25]. Conversely, an early Arab period of agricultural expansion in the Messara Plain, located ca. 120 km east to Asi Gonia, is inferred from an increase in flood events in the Anapodaris Gorge [71]. At the Asi Gonia peat bog, during the Arab period, local agro-pastoral indicators are absent and fire frequency decreased, while the landscape was more open than ever (Fig 8). The lack of evidence of anthropogenic impact at a time when human activities such as beekeeping or wood exploitation for timber and shipbuilding are well documented in Crete [25, 72], highlights the limitations of paleoecological archives.

The 2^nd^ Byzantine period corresponds to a time of change towards local drier conditions. Around 1000 AD, grazing and fire indicators increased anew and remained particularly high for ca. 150 years, while shrubs and trees decreased.

After the short decline in human pressure during the second half of the 12^th^ century, the change to Venetian domination did not alter the openness of the landscape. Fire frequency was moderate and more irregular than before, while fire magnitude increased. Dung fungal spores accumulation rates were not high but constant.

From the Venetian period onward, historical archives of economic activities and population have become more numerous [26]. From the 13^th^ to the 20^th^ century, trends in anthropogenic impact on the AG11 sequence are consistent with historical records and even mirror the demographic estimates for the last 500 years ([25], Fig 8). Basically, the Venetian rule resulted in the strongest anthropogenic impact, with a maximum in wild grass and weeds that is synchronous with low representation of evergreen oak. Dung indicators do not change significantly with respect to the 2^nd^ Byzantine period and peaks are associated with fire episodes. At the local scale, archeological investigations suggest that Kallikratis, the hamlet located less to 2 km west of the peat bog, was occupied at least since Venetian times [72].

A short and large magnitude event disrupted the deeply-anthropized Venetian period between 1350 and 1400 AD. Environmental proxies show an abrupt fall in herbaceous taxa and a rise of maquis species. No fire episodes are recorded at this time, local humidity reached a maximum and SAR increased meaningfully (Fig 8). This episode very likely corresponds to the first hit of Black Plague that struck southern Europe and killed one half of the Cretan population [25]. Following this event, a high magnitude charcoal peak followed by an increase in anthropogenic pollen indicators and dung fungal spores at around 1400 AD is the local illustration of the use of fire to reconquer land for agro-pastoral purposes [4].

The Ottoman period corresponds to less human pressure on the peat bog and the onset of a fire regime characterized by rare and low magnitude events. Since the beginning of the 19^th^ century, the demographic curve and the fluctuations in anthropogenic impact recorded in the Asi Gonia AG11 sequence have had opposite trends. The strong population decline caused by plagues and wars corresponds to a new grazing phase (Fig 8 and [25]). It is possible that this part of the White Mountains represented a refuge for local communities. Conversely, the prosperity and population increase characteristic of the late Turkish period (after 1850 AD) do not match the low human impact recorded locally. The local populations may have been somehow disconnected from the coasts where urban populations were growing rapidly (Fig 8)

Throughout the Asi Gonia record, several pieces of evidence point to other forms of land use different than grazing (Fig 8). The three key trees associated with the anthropization of the Mediterranean (i.e., *Olea*, *Juglans* and *Castanea* (the OJC group, [73]), were present until the Roman period. Chestnut and walnut appeared at ca. 150 AD, just after the first grazing pressure decrease, along with a rise in *Olea*. In Asi Gonia, local olive cultivation is difficult to infer from pollen data alone. *Olea* pollen, which does not exceed 5%, may have originated from wild olive trees and/or from extra local populations. The current elevation-limit for olive tree cultivation is ca. 600 m elevation. Given that *Olea* pollen percentages never reached modern values, we infer that olive trees were not likely to have been locally cultivated at higher elevations in the past. The Turkish administration promoted olive-oil production [26] but it does not seem to have reached the eastern edge of the White Mountains.

The continuous presence of low-pollinizer *Juglans* from 100 AD to 650 AD suggests walnut was cultivated in this part of Crete during Roman and 1^st^ Byzantine times, which is consistent with the hypothesis of its Roman introduction in Crete [24]. *Castanea* cultivation is also clear, particularly during the Byzantine 650–800 AD period [24]. Chestnut trees have persisted since then in the area. Grapevine is commonly cultivated up to 900 m elevation in Crete. However, the *Vitis* pollen curve at Asi Gonia peat bog may be related to the wild subspecies *Vitis vinifera*-*sylvestris*, which often grows on plane trees, in as much as the overall low amounts of *Vitis* pollen only rise when *Platanus* pollen increases (e.g. 350–450 AD).

Cultivation of crops is registered from the beginning of the sequence but indicators remain scarce. Some periods seem to experience more important and/or local cereal cultivation, with the regular presence of *Triticum*-type pollen, which is the least ambiguous pollen type among cereals [51]: 1) the first century AD; 2) around 950 AD, at the transition from the Arab to the 2^nd^ Byzantine period and again between 1000 and 1050; 3) around 1500 and 1620 during the Venetian time; 4) around 1750 during Turkish domination and 6) present (Fig 5).

Finally, data from the last 50 years are consistent with the modern Cretan rural economy: the *Olea* peak reflects olive production on slope terraces developed after the Second World War. Additionally, the recent rise of grazing indicators may be due to the political attempts of the European Community to encourage herding and olive production since the 1980s [25].

### Ecological trajectories and tipping points

Pollen data suggest that during the last 2000 years, the vegetation history of Asi Gonia was characterized by three main periods: 1) a first one dominated by evergreen oak forest (until ca. 850 AD); 2) followed by heather maquis (ca. 850 to 1870 AD); 3) and finally by very open landscape with dwarf-shrubs such as *Sarcopoterium* and *Olea* at a regional scale (ca. 1900 AD to the present; Figs 5 & 8). Atherden & Hall (1999) interpreted the reconstructed evolution of evergreen oak woodland toward the current phrygana as evidence supporting "the progressive model of human impact" (*sensu* Oldfield, 1983; i.e. non-reversible vegetation changes over time). Although our data also suggest a long-term linear succession of vegetation communities, the process seems to be more complex, scale-dependent and characterized by rapid changes associated with tipping-points in ecosystem dynamics.

During the first phase of vegetation development (before 850 AD), deciduous oak and other tree species currently present in the woodlands of the Cretan mountains, e.g. maple (*Acer*) and cypress (Cupressaceae) [24, 25], were poorly represented (Fig 5). High percentages of evergreen oak pollen, along with maquis species (i.e., *Erica*-type (mainly originating from heather-tree), *Arbutus*, *Cistus* and *Pistacia*) that were already abundant during the first 4 centuries, suggest that sclerophyllous woodland dominated the watershed. This plant association is likely a secondary formation inasmuch as prickly-oak is highly resilient to both grazing and burning and may dominate holm oak communities [24, 25].

PCA analysis suggests that, during the Roman and first half of the 1^st^ Byzantine periods, burning and grazing management had a reversible impact on vegetation, given that its composition oscillated around a theoretical equilibrium point (Fig 8). These centennial-scale dynamics are consistent with Oldfield’s cyclical model of human occupation (1983). Thus, the levels of anthropogenic impact and associated practices at the time allowed prickly-oak and arbutus-heather formations to regenerate after disturbance, as observed during the 1980’s by Rackman & Moody [25] in different mountain areas of Crete. Palynological richness increased as mean fire magnitude decreased in the transition from the Roman to the 1^st^ Byzantine period at ca. 300, and reached a maximum when the local grazing and burning were lowest (ca. 500–600 AD; Fig 8). The second half of the 1^st^ Byzantine period (ca. 600 AD to 850 AD) featured a transition from early evergreen oak woodland to health.

A first tipping-point was reached at ca. 850 AD: pollen data show a sharp increase in heather pollen at expense of evergreen oak pollen, suggesting a shift from sclerophyllous woodland to maquis (Fig 8). At that time, high fire frequency correlated with high-magnitude fire episodes would suggest that fire, likely set by humans, may have led to this vegetation shift. Arbutus-heather maquis dominated the landscape from 850 AD to 1900 AD. Shifts in dominance from maquis to recovering evergreen oak occurred, especially during the 12^th^ century and from 1650 to 1900 in association with changes in human pressure.

According to pollen taxa richness analysis (Fig 8), land use seems to have helped maintain floristic biodiversity, especially when human agro-pastoral impact intensified during Venetian domination ca. 1400 AD. As in the previous phase of vegetation development, community composition oscillated at centennial scales in accordance with the cyclic model of human occupation. This pattern suggests that reversible (cyclic) centennial-scale shifts in vegetation were embedded in a long-term trend of irreversible (progressive) change. Such scale-dependency of human impact on ecosystem processes has been proposed for other Mediterranean sites (e.g., [74]).

A second tipping-point in ecosystem dynamics occurred at the beginning of the 20^th^ century (Fig 8) and was associated with a shift from maquis to present-day phrygana. Between ca. 1850 and 1900, combined heather and evergreen oak pollen dropped from 70 to 20% of the TLP. This decrease was accompanied by a rise of *Sarcopoterium*, asphodel and other phrygana and/or steppe indicators along with an unprecedented rise of *Olea*. The absence of grazing indicators and negligible biomass burning suggest that the drivers of this change are possibly related to regional crop intensification and other economic activities. Charcoal making, for instance, was an attested practice until the 1960s in the mountains above Asi Gonia [24] that could have contributed to the change in vegetation composition. However, present-day local maintenance of phrygana is probably linked to sheep and goat grazing inasmuch as the western Cretan mountains have been overgrazed and exposed to uncontrolled practices since the 1980s [75]. Our data thus show that the ecological changes starting at the beginning of the 20^th^ century are unprecedented and associated with a pronounced loss of pollen taxa biodiversity (Figs 5 and 8).

## Conclusion

Understanding the processes that led to the recent evolution of the Mediterranean landscape is a challenging question that can be addressed with paleoecological data. In this sense, the new Asi Gonia record, with a robust chronological control and multiproxy analyses, highlights land-use history and ecosystem change during 2000 years. The record provides new insights on the origins of the peat bog. The accumulation of sediments began during the transition from the Hellenistic to the Roman period. At the time, the area was already dedicated to grazing and fire was largely used for the maintenance of sylvo-agropastoral areas. We propose that human-induced processes associated with hydrological disturbance in the watershed gave rise to the bog.

During the two last millennia, vegetation dynamics have been strongly linked to human history. Pastoralism, either alternated or combined with other human practices, has been the dominant economic activity. Some practices, such as crop cultivation, can be reconstructed from our data. In other cases, such as wood exploitation, information needs to be drawn from the historical and archeological record. The long-term importance of breeding in the Cretan mountains is well-known [25]. In this study, the use of dung fungal spores attests to local grazing since at least the early Roman period. Pastoral practices were roughly stable during six centuries and changes in practices probably occurred thereafter.

Multiproxy analyses show that the peatland is impacted by local human activities inasmuch as the main sedimentological changes are not correlated with those in the surrounding landscape. The scarcity of regional paleoclimate studies with high temporal resolution for the Late Holocene [18, 20] makes it difficult to accurately link the evolution of Asi Gonia peat bog to Eastern Mediterranean climate changes. While a clear connection exists between local hydroclimatological change and a shift to more humid conditions at around 500 AD, after 850 AD, human-induced changes are likely to have prevailed.

Although breeding was, and still is, of great local importance, the major tipping points in ecosystem trajectories are not associated with grazing pressure. Increased knowledge of the local occupation history might permit a better understanding of the processes leading to abrupt change. Thus, archeological characterization and dating of the numerous remains found in the watershed are crucial.

The shaping of the modern landscape did not culminate until the 20^th^ century and is associated with pollen, fern and fungal spore biodiversity loss at the scale of both the surrounding landscape and the wetland. In their book *The making of the Cretan landscape*, which is the main synthesis on the subject and inspired the title of this paper, Rackham & Moody [25] conclude that “Cretan vegetation is resilient. The idea of progressive deforestation, on which “Ruined Landscape” theory depends, has been falsified”. Unfortunately, our case study does not support their conclusions. We might be less optimistic at the onset of the 21^th^ century, but our results suggest that the present state of the ecosystem has no analog in the past. In addition, the dramatic drying of the peat bog seems hardly reversible under the combined influence of global drying and spring-capture. Thus, an exceptional man-made wetland will be destroyed by man.
